# Fine-Scale Genetic Response to Landscape Change in a Gliding Mammal

**DOI:** 10.1371/journal.pone.0080383

**Published:** 2013-12-26

**Authors:** Ross L. Goldingay, Katherine A. Harrisson, Andrea C. Taylor, Tina M. Ball, David J. Sharpe, Brendan D. Taylor

**Affiliations:** 1 School of Environment, Science and Engineering, Southern Cross University, Lismore, New South Wales, Australia; 2 Australian Centre for Biodiversity, School of Biological Sciences, Monash University, Victoria, Australia; 3 Central Queensland University and Queensland Parks and Wildlife Service, Mackay, Queensland, Australia; Università degli Studi di Napoli Federico II, Italy

## Abstract

Understanding how populations respond to habitat loss is central to conserving biodiversity. Population genetic approaches enable the identification of the symptoms of population disruption in advance of population collapse. However, the spatio-temporal scales at which population disruption occurs are still too poorly known to effectively conserve biodiversity in the face of human-induced landscape change. We employed microsatellite analysis to examine genetic structure and diversity over small spatial (mostly 1-50 km) and temporal scales (20-50 years) in the squirrel glider (*Petaurus norfolcensis*), a gliding mammal that is commonly subjected to a loss of habitat connectivity. We identified genetically differentiated local populations over distances as little as 3 km and within 30 years of landscape change. Genetically isolated local populations experienced the loss of genetic diversity, and significantly increased mean relatedness, which suggests increased inbreeding. Where tree cover remained, genetic differentiation was less evident. This pattern was repeated in two landscapes located 750 km apart. These results lend support to other recent studies that suggest the loss of habitat connectivity can produce fine-scale population genetic change in a range of taxa. This gives rise to the prediction that many other vertebrates will experience similar genetic changes. Our results suggest the future collapse of local populations of this gliding mammal is likely unless habitat connectivity is maintained or restored. Landscape management must occur on a fine-scale to avert the erosion of biodiversity.

## Introduction

Habitat loss and fragmentation are viewed as two of the most serious threats to biodiversity worldwide [1–3]. Mitigating these twin impacts requires an understanding of the spatio-temporal scale at which they operate and knowledge of the ecological attributes of species most at risk. Until recently, ecological studies were the primary means by which the response of species to habitat loss and fragmentation were investigated (e.g. [4–7]). Whilst this approach has proven powerful it relies heavily on local population collapse having occurred in some but not all habitat remnants after many decades of exposure, by which time it may be too late to reverse or alter the path of landscape change. In contrast, population genetic approaches can identify the symptoms of population disruption in advance of population collapse. Such an approach has recently identified significant population genetic changes at small spatio-temporal scales in common vertebrates as a consequence of intense urbanisation [8]. 

Isolation and loss of available habitat lead to reduced gene flow and small populations. The extent that populations become functionally isolated (i.e. for dispersal) depends on a number of factors, including the availability of habitat corridors, the composition of the landscape matrix in which remnants are embedded and a species’ vagility (i.e. its ability to use or cross the matrix) [9 -11]. The landscape matrix is an altered but heterogeneous environment containing residual, regenerating or exotic habitat components that may promote or hinder the movement of species (e.g. [11–13]). It may also contain human-constructed elements such as roads that may further reduce or prevent the movement of species [8,14]. 

 The ability to cross the matrix is unknown for most species [11]. However, such information is fundamental to devising appropriate conservation programs [13]. For example, increasing the quality of the matrix may offer an effective alternative to providing connecting corridors [11,15]. Studies that examine how a species responds to the matrix have relied on direct observation of animals that have been experimentally displaced, such that they are forced to disperse and reveal their preference or ability to use elements of the landscape matrix (e.g. [11,16,17]). Although these studies provide important insights, physical dispersal does not always equate to gene flow, with migrants frequently experiencing reduced reproductive success owing to poor body condition [18,19]. Genetic studies can inform whether predicted behavioural responses align with genetic measures of connectivity or isolation (e.g. [20,21]). 

Genetic theory predicts that small, genetically-isolated populations will be subject to inbreeding and loss of genetic variation through drift. Increased levels of inbreeding can give rise to inbreeding depression, leading to reduced fitness and an increased extinction risk [22]. Erosion of genetic diversity will place populations at higher risk of extinction by reducing their capacity to evolve in response to environmental change [22–25]. Thus, landscapes should be managed to minimise negative genetic effects (i.e. loss of genetic diversity, inbreeding depression and loss of evolutionary potential) where the conservation of biodiversity is a priority. Consequently, knowledge of the scale at which these impacts occur should be central to such management.

Tree-dependent ground-avoiding mammals are predicted to be sensitive to habitat loss and fragmentation. We chose the squirrel glider (*Petaurus norfolcensis*), an Australian gliding mammal dependent on tree cover for movement [26–28], to examine how a species responds to habitat loss and fragmentation associated with urban and agricultural development. It is listed as endangered in the southern part of its range. The squirrel glider occupies landscapes historically subjected to clearing for agriculture [29]. Where its range extends along the coast in eastern Australia, these landscapes are now subject to urbanisation [30]. The squirrel glider can provide an important case study because, by virtue of having a broad geographic range, it is possible to examine independent landscapes that are following a similar trajectory of landscape change. We chose two landscapes, approximately 750 km apart, where an earlier pattern of clearing for agriculture is being followed by further clearing and fragmentation for housing and roads. Our previous work has demonstrated that where some degree of tree cover is retained, populations may show minimal genetic structure over very large spatial scales (>1000 km) [31]. However, where tree cover has been removed for a long period (>50 years) a genetic signature of isolation can manifest itself. Here we examine genetic structure and diversity over smaller spatial (1-50 km) and temporal scales (20-50 years). Within both landscapes we chose sample locations that were fully isolated by urban development, locations separated by clearing for agriculture or urbanisation over small distances, and locations that still appeared connected by tree cover. We predict that genetic structuring will be independent of geographic distance because the presence or absence of tree cover between our sampling locations will be independent of distance. Furthermore, we predict those locations with reduced gene flow should show reduced genetic diversity and higher levels of genetic relatedness (indicative of inbreeding). 

## Materials and Methods

### Ethics statement

This research was conducted under permits issued by the Queensland Parks and Wildlife Service (WISP00170002/WISP02155504/WITK01317804). Samples were collected with institutional animal ethics approval (Southern Cross University 02/2, 03/4, 03/10, 04/7, 04/28, 05/24; Central Queensland University 03/06–141) in accordance with the Australian Government National Health and Medical Research Council’s Code of Practice for the Care and Use of Animals for Scientific Purposes. 

### Study areas

The city of Mackay is located in tropical northern Queensland and has a population of 120,000 people. Over 50% of the vegetation had been cleared for sugar cane production and cattle grazing. Our sample locations included sclerophyll woodland remnants as well as large (>5000 ha) blocks of woodland. Some remnants (e.g. Mt Vince) included vegetation such as rainforest that is not used by squirrel gliders. The remnants had been in their current configuration for at least 15 years. 

The city of Brisbane is located in subtropical southern Queensland and has a population of 1 million people. It has experienced clearing over a long period of time but urban development has intensified in the last 30 years. Several large motorways and freeways now traverse the city and disconnect many areas of remnant forest from each other [30,32]. Our sample locations included remnants of dry sclerophyll forest. One (Bracken Ridge) was located on the north side of Brisbane, and north of the Brisbane River, while the others were located to the south. All are now surrounded by urban development and have been in this configuration for at least 20 years.

### Sample collection

Squirrel gliders were caught in traps placed on the trunks of trees or were removed from nest-boxes, between 2003 and 2005 in Mackay and between 2002 and 2006 in Brisbane. In Mackay, animals were trapped at 12 locations (see 27,33 for details). In Brisbane, trapping was conducted at Minnippi Parklands, Belmont Hills, Mt Petrie, Bracken Ridge, Karawatha and Kuraby ([34,35], Goldingay unpublished data; Sharpe unpublished data). Animals were also sampled from within 3-15 nest boxes established at Minnippi Parklands, Minnippi East, Belmont Hills and Gateway. Although nest-box groups are likely to contain multiple family members [36] we sampled all encountered individuals for this study in order to maximise our chances of genetic detection of dispersal. Gateway was the only location where we relied solely on nest box captures so it is unlikely to have biased our estimated allele and genotype frequencies and consequently estimates of population differentiation (nonetheless, see precautions described below in genetic analyses). Ear tissue samples (1-2 mm wide) were taken from captured individuals and stored in 95% ethanol prior to DNA extraction.

### Genotyping

We extracted genomic DNA from ear tissue following the methods of Sunnucks & Hales [37]. Samples were genotyped for six microsatellite loci: Pn16 and Pn49 (developed for squirrel gliders [38]) and Pet1, Pet6, Pet7 and Pet9 (developed for the closely-related sugar glider (*P. breviceps*) [39]). Microsatellite alleles were detected by electrophoresis on 6% polyacrylamide sequencing gels and either autoradiography (Pn16, Pn49) or using a LI-COR Global IR2 two-dye DNA sequencer (model 4200; Pet1, Pet6, Pet7 and Pet9). The Bracken Ridge, Karawatha, Cape Hillsborough, Kinchant and Padaminka genotypes included here formed part of the broadscale analysis of Taylor et al. [31].

### Genetic analyses

We used the individual-based Bayesian spatial clustering algorithm implemented in TESS 2.3.1 [40] to identify genetic clusters within our samples. TESS was run using the CAR admixture model (100 iterations of 10^6^ sweeps, discarding the first 30,000), with the spatial interaction parameter set to 0.6, and the number of genetic clusters (*K*) set from 2-12. We analysed our two geographic regions separately to determine the number of clusters (*K*) present. The most likely value of *K* was chosen as the point of greatest change in a plot of mean DIC (Deviance Information Criteria) across 100 runs versus *K*. Average cluster probabilities (population Q values) across 10 runs with the lowest DIC for the most likely *K* were determined using CLUMPP [41], and plots produced in DISTRUCT [42] based on CLUMPP output.

We used GENALEX 6 [43] to calculate Queller & Goodnight’s [44] pairwise relatedness among individuals. We generated upper and lower 95% confidence intervals from 1000 random reshufflings of values among populations to test whether the mean observed relatedness for a population was greater than that expected under the null hypothesis of no difference among populations. We used FSTAT 2.9.3 [45] to calculate gene diversity (Hs) and allelic richness (AR), a measure of allelic diversity that takes into account differences in sample sizes by standardising to the smallest number of individuals typed for a locus in a sample (separately for regions) [46]. We calculated pairwise *F*_ST_ values as a measure of genetic differentiation among populations and assessed their significance using 1000permutations. These were converted to *F*_ST_ /(1 - *F*_ST_) and used along with Euclidean (log-transformed) distances among sampling sites to examine isolation-by-distance for the two regions separately, via Mantel tests performed using the web version of GENEPOP [47,48] with 100 permutations. To provide an index of relative genetic isolation for a population we calculated its average *F*_ST_ across all its pairwise *F*_ST_ values (within the relevant region).

As population samples derived from nest box animals are likely to contain relatives, genetic estimates of that population’s degree of isolation might be upwardly biased. To test whether this was the case for the highly genetically distinct nest box-sampled Minnippi Parklands, we randomly sub-sampled 20 individuals from this location and repeated all of the above analyses. The results did not differ from those obtained when all sampled individuals were included.

### Contemporary gene flow

We used BayesAss 1.3 [49] to investigate migration among our sample locations. This program uses individual multilocus genotypes to estimate recent (1-3 generations) migration rates between location pairs (e.g. [50,51]). It uses Markov Chain Monte Carlo (MCMC) techniques to estimate posterior probabilities for the proportion of individuals in each sample location that are assigned to other populations. We analysed our data using MCMC runs of 3 000 000 iterations to ensure convergence. We used the first 999 999 iterations as our burn-in and used a thinning interval of 2 000 iterations. We used delta values of 0.15. The estimated values were compared to those that occur for a given number of sample locations when there is no information in the data.

## Results

### Analysis of genetic structure

####  Mackay Landscape

**Figure 1:**
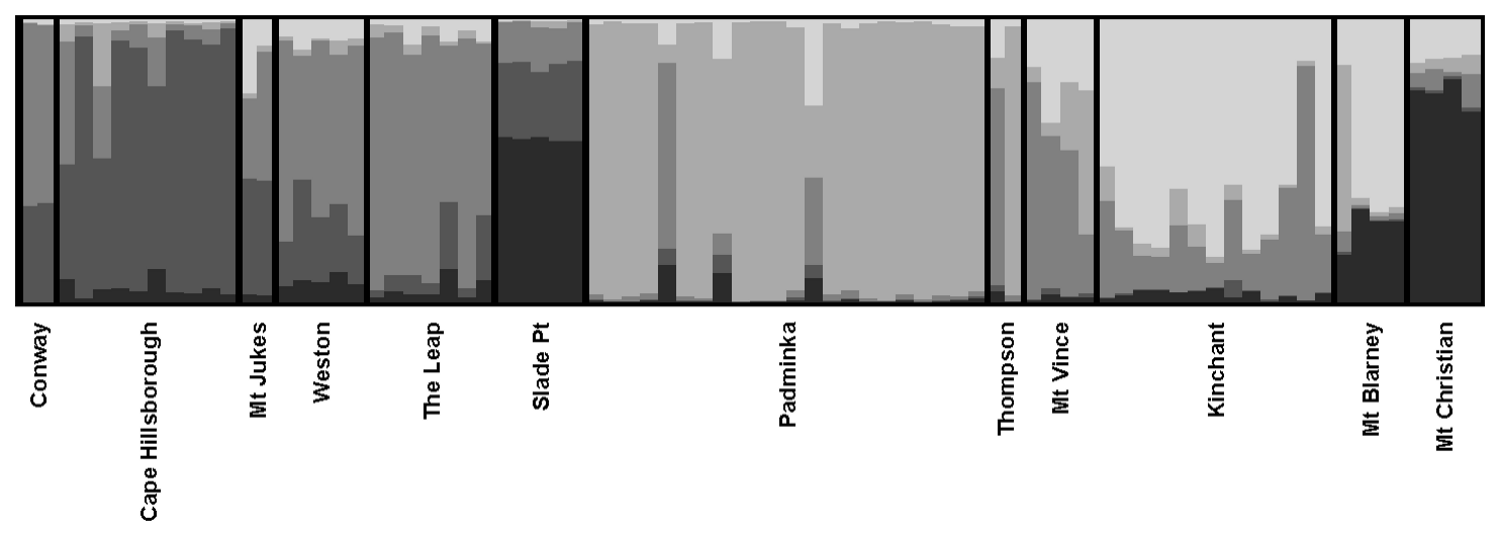
Genetic clustering analysis for Mackay (K=5) samples. Different grayscales represent the five different genetic clusters. Individuals are grouped by location and are represented by columns and the proportion of each grayscale in a column represents their proportional assignment to a cluster. Locations are arranged north to south (left to right).

The TESS analysis of 80 samples from 12 locations suggested the most likely number of genetic clusters was five (Figure 1). Some locations were strongly associated with a single cluster, which suggests genetic isolation. Individuals from Padaminka were predominantly assigned to cluster 4 (average Q=0.88), individuals from Cape Hillsborough predominantly to cluster 2 (Q=0.78), individuals from The Leap predominantly to cluster 3 (Q=0.78) and individuals from Mt Christian predominantly to cluster 1 (Q=0.74). Locations within 3.5 km of The Leap (Weston) and Padaminka (Thompson’s) had membership predominantly (Q=0.60, Q=0.53, respectively) in the same cluster as their nearest neighbour. In contrast, Kinchant had membership predominantly in cluster 5 (Q=0.66) which was different to Padaminka, its nearest neighbour 8 km away. 

#### Brisbane Landscape

**Figure 2:**
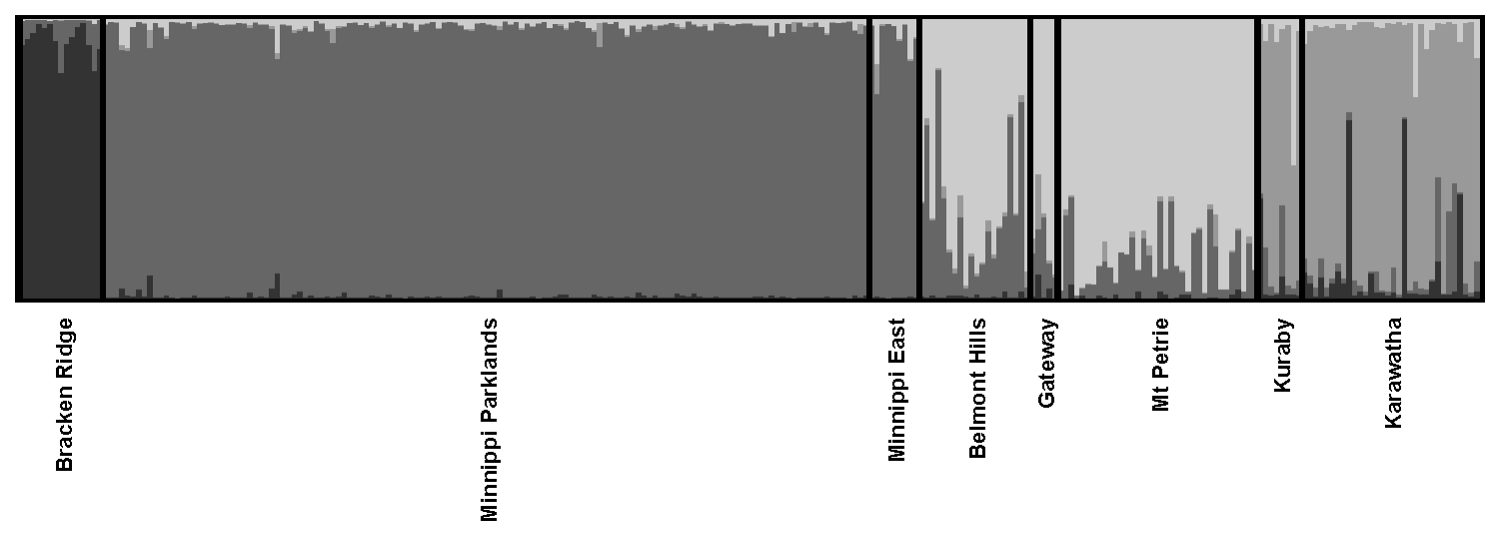
Genetic clustering analysis for Brisbane (K=4) samples.

The TESS analysis of 265 samples from eight locations suggested there were four genetic clusters (Figure 2). Individuals from Bracken Ridge were predominantly assigned to cluster 1 (Q=0.93), individuals from the two Minnippi locations to cluster 2 (Q=0.97 and 0.92), individuals from Kuraby and Karawatha to cluster 3 (Q=0.76 and 0.82), and individuals from the remaining three locations (Belmont Hills, Mt Petrie, Gateway) that were within 3 km of each other predominantly to cluster 4 (Q=0.68-0.84). The very high Q values for the Minnippi sites suggest isolation from the nearest location (Belmont Hills) just 3 km away. 

### Genetic diversity

**Figure 3:**
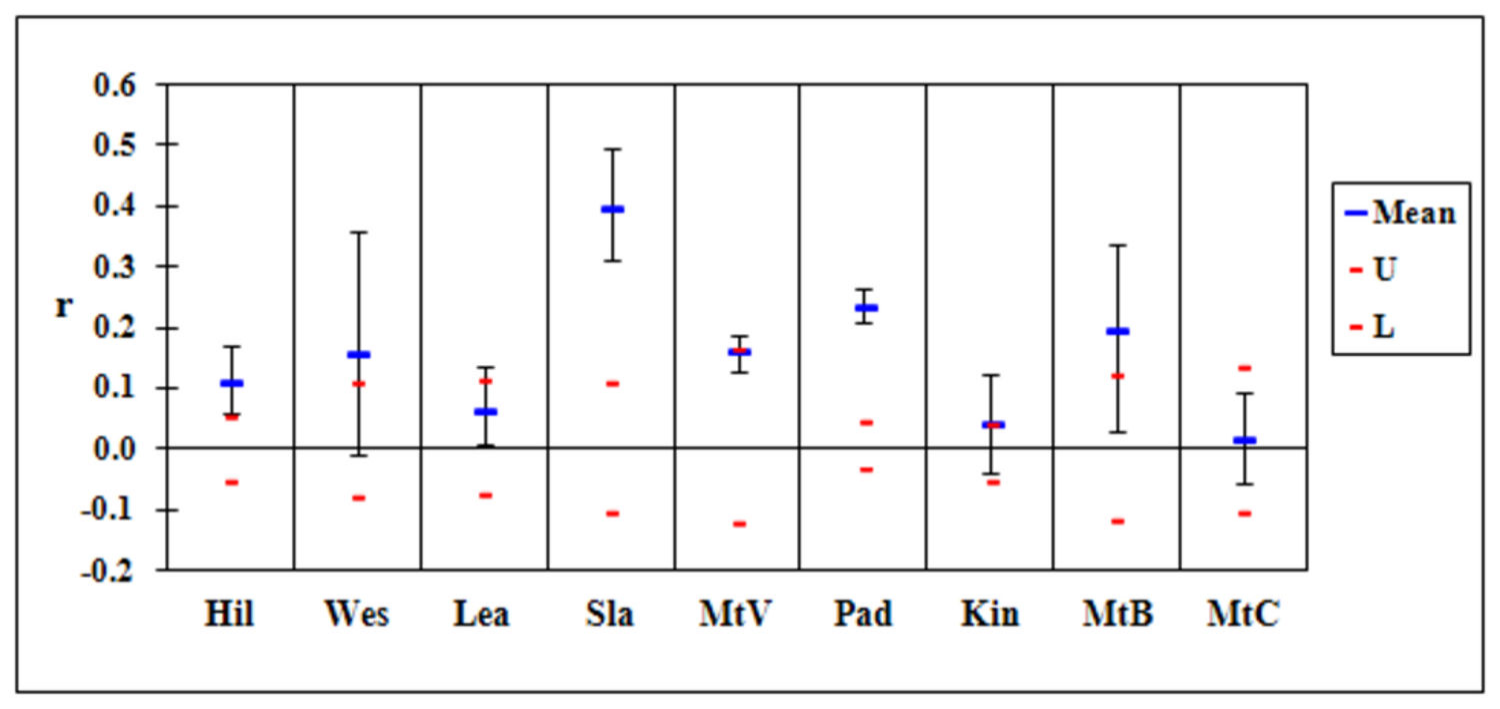
Mean relatedness for sample locations in Mackay. Location names are abbreviated as in Table 1. The red lines indicate the upper (U) and lower (L) 95% confidence interval expected for that population under the null hypothesis of no difference among populations. Mean values above that interval for Hil, Sla and Pad indicate relatedness is higher than expected.

Genetic diversity (Hs) in the Mackay region was low at the location (Padaminka) that formed the tightest genetic cluster (Table 1 and Figure 3). In contrast, locations that were part of a broader cluster group (The Leap, Mt Christian) showed higher genetic diversity. In Brisbane, the Minnippi cluster had the lowest genetic diversity, while those locations with the lower cluster values had the highest genetic diversity (Mt Petrie, Karawatha) (Table 1 and Figure 2). These results were also reflected in the measure of allelic richness. Remnant area also influenced genetic diversity. We compared Hs for large (>450 ha) and small (<200 ha) remnants. The mean values of these groups (large = 0.91±0.01; small= 0.82±0.02) were significantly different (*t*=4.06, d.f.=10, *P*=0.002). 

**Table 1:** Genetic diversity and differentiation parameters for squirrel glider sample locations.

**Location**	**Abbrev.**	**Area (ha)**	**Age (yrs)**	**N**	**Hs**	**AR**	**Avge *F*_ST_**
**Mackay** (AR for n=3)							
1 Cape Hillsborough	Hil	>500	30	10	0.87	4.48	0.097
2 Mt Jukes	–	5000	0	2	–	–	–
3 Weston	Wes	150	5-15	5	0.85	4.31	0.088
4 The Leap	Lea	300	5-15	7	0.88	4.52	0.075
5 Slade Point	Sla	215	50-60	5	0.68	2.80	0.172
6 Mt Vince	MtV	25	20-30	4	0.84	4.09	0.096
7 Thompsons	–	100	30-40	2	–	–	–
8 Padaminka	Pad	65	30-40	22	0.78	3.90	0.148
9 Kinchant	Kin	485	20-30	13	0.91	4.77	0.069
10 Mt Blarney	MtB	95	15-20	4	0.83	4.08	0.122
11 Mt Christian	MtC	>600	0	4	0.94	4.88	0.078
**Brisbane** (AR for n=5)							
1 Bracken Ridge	Bra	100	30-50	15	0.85	5.71	0.090
2 Minnippi Parklands	MnP	50	30-50	138	0.75	4.59	0.094
3 Minnippi East	MnE	25	30-50	9	0.67	3.93	0.141
4 Belmont Hills	Bel	110	30-50	22	0.86	6.17	0.069
5 Gateway	Gat	700	30-50	5	0.88	6.50	0.068
6 Mt Petrie	MtP	700	30-50	36	0.91	7.02	0.056
7 Kuraby	Kur	140	20-30	8	0.90	6.68	0.063
8 Karawatha	Kar	750	20-30	32	0.92	7.45	0.051

Sites are arranged north to south. Age= the number of years before sampling when the remnant became isolated from other habitat areas. N = sample size, Hs = gene diversity, AR = allelic richness (standardised to a minimum sample size of either 3 or 5). Average *F*_ST_ was calculated by averaging all pairwise *F*_ST_ values (within the relevant region) involving that population.

### Genetic differentiation among populations

Pairwise *F*_ST_ values were generated for each population (Table 2). This revealed that 29 of 36 comparisons between locations within Mackay were significant. Padaminka, Slade Pt, Kinchant and Cape Hillsborough were each significantly diverged from all other locations. Divergence was not significant among Mt Vince, The Leap, Weston and Mt Christian. In Brisbane, significant divergence was detected among most locations except the neighbouring locations of Mt Petrie and Gateway, and Karawatha and Kuraby. Gateway also did not diverge significantly from Karawatha and Kuraby. The broad level of differentiation is evident in the average *F*_ST_ values for a location (Table 1). As predicted, we detected no evidence of an isolation-by-distance effect in either Mackay or Brisbane (*P* = 0.52 and 0.23, respectively).

**Table 2:** Pairwise *F*_ST_ values (below diagonal) and Euclidean distances (km) (above diagonal) among sample locations for the two study landscapes.

**Mackay**	Hil	Sla	Lea	Wes	Kin	Pad	MtV	MtB	MtC
Hil		26.7	16.5	13.7	37.8	29.4	29.3	60.8	82.8
Sla	**0.162**		19.9	19.3	37.2	19.6	26.5	39.0	59.8
Lea	**0.070**	**0.145**		3.0	22.0	13.5	13.4	46.5	68.0
Wes	**0.070**	**0.146**	0.019		25.3	15.8	16.3	48.0	69.8
Kin	**0.070**	**0.138**	**0.038**	**0.046**		17.1	10.0	40.5	61.3
Pad	**0.141**	**0.227**	**0.139**	**0.157**	**0.112**		8.0	31.6	55.3
MtV	**0.097**	**0.181**	0.047	0.079	**0.057**	**0.123**		36.1	59.7
MtB	**0.113**	**0.225**	**0.100**	**0.137**	**0.057**	**0.154**	**0.107**		23.7
MtC	**0.056**	**0.153**	0.041	0.051	**0.032**	**0.132**	0.081	0.082	
**Brisbane**	Bra	MnE	MnP	Bel	MtP	Gat	Kar	Kur	
Bra		17.2	16.3	20	24.2	22.1	31	30.2	
MnE	**0.180**		0.8	2.6	6.5	4.4	15.1	14.2	
MnP	**0.151**	**0.065**		2.9	7.1	4.7	15.2	14.3	
Bel	**0.078**	**0.163**	**0.115**		3.3	2.4	12.1	11.2	
MtP	**0.065**	**0.121**	**0.106**	**0.029**		2.2	10.6	9.9	
Gat	**0.067**	**0.173**	**0.122**	**0.040**	0.015		11.5	10.8	
Kar	**0.043**	**0.131**	**0.104**	**0.033**	**0.026**	0.018		0.05	
Kur	**0.051**	**0.157**	**0.118**	**0.039**	**0.028**	0.045	0.003		

Significant *F*_ST_ values (P<0.05) are shown in bold.

### Pairwise relatedness within locations

**Figure 4:**
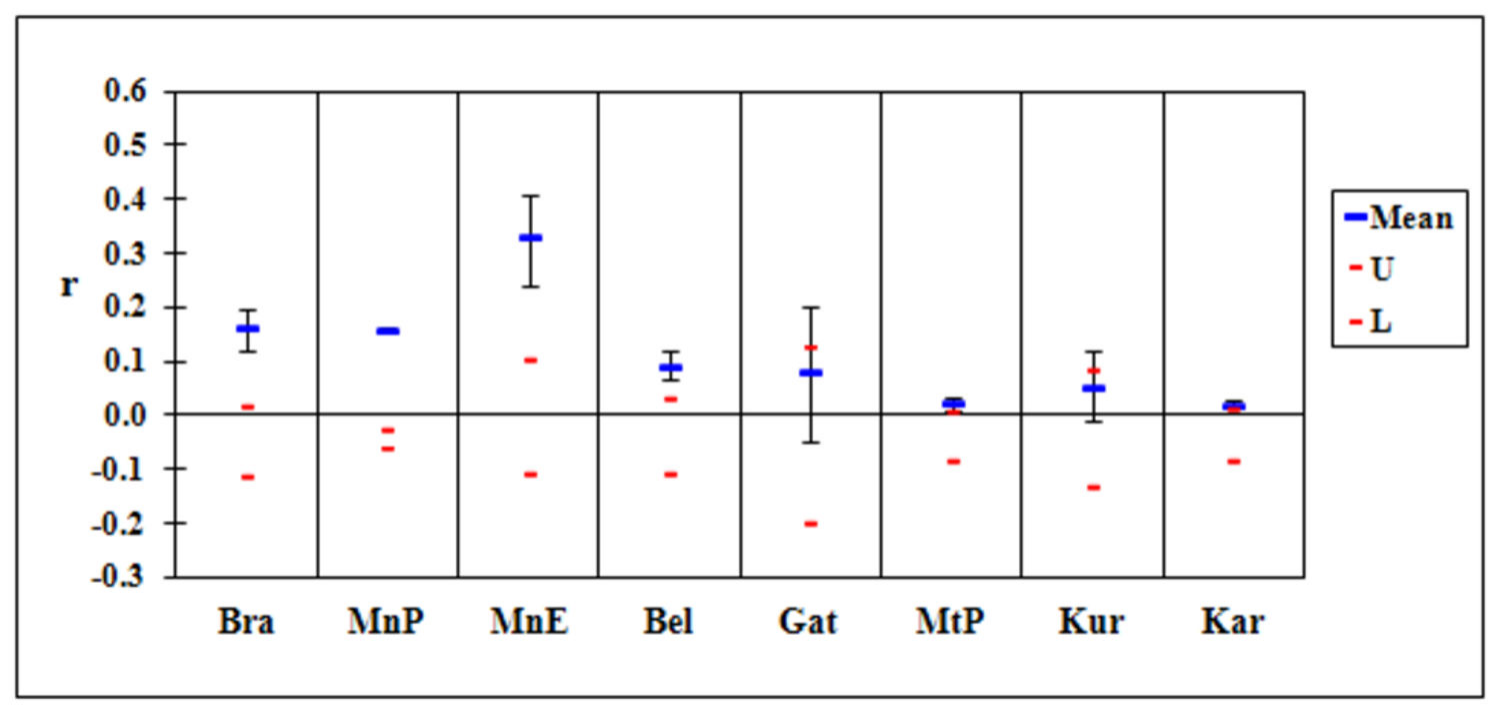
Mean relatedness for sample locations in Brisbane. Mean values above the red lines for Bra, MnP, MnE and Bel indicate relatedness is higher than expected.

The locations at Mackay (Slade Pt, Padaminka) with the lowest genetic diversity were also the only ones that had significantly elevated mean relatedness (the means were outside the simulated confidence intervals; Figure 3). A similar result was obtained in Brisbane (Figure 4) with the isolated locations (Bracken Ridge, both Minnippi sites) having much higher than expected measures of relatedness. Relatedness was also higher than expected among individuals sampled from Belmont Hills. 

### Contemporary gene flow

In the BayesAss analysis most of our sample locations showed very low migration rates with values no different to those providing no information (Table 3). The exception in Mackay was a migration rate from The Leap to Padaminka of 0.270. The exception in Brisbane was a migration rate of 0.225 from Minnippi Parklands to Minnippi East. These migration rates were uni-directional, with those estimated in the reverse direction being uninformative. BayesAss may provide reasonably accurate estimates of migration rate if genetic differentiation occurs at a level of *F*_ST_ ≥0.05 [52]. The reliability of the estimates will also be influenced by the number of individuals sampled, the number of loci and the number of alleles per locus [52]. The level of genetic differentiation (*F*_ST_≥0.05) documented for populations in both landscapes suggests that these estimates of recent migration rate are likely to be reasonably accurate [52].

**Table 3:** Contemporary migration rates estimated using BayesAss 1.3.

**Recipient (N)**	**Source**								
**Mackay**	Hil	Sla	Lea	Wes	Kin	Pad	MtV	MtB	MtC
Hil (10)	**0.885**	0.020	0.010	0.017	0.015	0.014	0.012	0.012	0.015
Sla (5)	0.013	**0.875**	0.011	0.015	0.035	0.013	0.013	0.012	0.012
Lea (7)	0.018	0.019	**0.806**	*0.019*	*0.076*	0.015	*0.018*	0.014	*0.016*
Wes (5)	0.026	0.026	*0.022*	**0.727**	*0.111*	0.021	0.024	0.021	0.023
Kin (13)	0.005	0.006	*0.008*	*0.006*	**0.951**	0.005	0.006	0.007	*0.006*
Pad (22)	0.007	0.006	***0.270***	0.006	0.006	**0.687**	0.006	0.007	0.006
MtV (4)	0.021	0.024	*0.064*	0.021	0.039	0.022	**0.765**	0.021	0.021
MtB (4)	0.018	0.025	0.034	0.024	0.026	0.031	0.026	**0.789**	0.027
MtC (4)	0.028	0.029	*0.016*	0.027	*0.026*	0.023	0.026	0.026	**0.780**
**Brisbane**	BR	MnE	MnP	Bel	Gat	MtP	Kar	Kur	
BR (15)	**0.978**	0.003	0.003	0.003	0.003	0.003	*0.003*	0.003	
MnE (9)	0.013	**0.697**	***0.225***	0.014	0.012	0.013	0.014	0.013	
MnP (138)	0.000	0.000	**0.998**	0.000	0.000	0.000	0.000	0.000	
Bel (22)	0.004	0.005	0.036	**0.931**	*0.004*	*0.011*	*0.005*	*0.005*	
Gat (5)	0.030	0.021	0.023	*0.050*	**0.714**	*0.112*	*0.031*	*0.020*	
MtP (36)	0.005	0.004	0.005	*0.031*	*0.004*	**0.939**	*0.008*	*0.004*	
Kar (32)	*0.041*	0.009	0.007	*0.006*	*0.009*	*0.029*	**0.865**	*0.033*	
Kur (8)	0.029	0.017	0.027	*0.026*	*0.015*	*0.055*	*0.115*	**0.717**	

Values represent migration from the source location listed across the top to the recipient location listed in the column at left. Simulations in BayesAss show that uninformative data will produce a mean migration rate of 0.021 (0.000-0.126, 95% CIs) for nine populations and 0.024 (0.000-0.134, 95% CIs) for eight populations. Values in bold and italics fall outside these intervals. Uninformative data for 8 and 9 populations produce a mean proportion of residents (diagonal values) of 0.833 (0.675-0.992, 95% CIs). Plain italicised values are pairs with *F*_ST_ <0.05.

## Discussion

### Genetic differentiation

Landscape change is now viewed as a leading cause of biodiversity loss [3,53–55]. However, the period of time and spatial scale at which populations are disrupted are poorly resolved. Our study has revealed highly genetically differentiated local populations of an arboreal mammal in two landscapes located 750 km apart. We identified significant genetic divergence over distances as little as 3 km and within 30 years (i.e. ~10 generations) of landscape change. Genetically isolated local populations experienced loss of genetic diversity, and significantly increased mean relatedness, suggesting increased inbreeding. These findings are consistent with those of Delaney et al. [8] who observed such effects in an urban landscape for three lizard species and a bird over similar spatio-temporal scales. 

Genetic analyses identified two key features of both our study landscapes. First, locations in Mackay (Padaminka) and in Brisbane (Bracken Ridge, Minnippi Parklands) showed pronounced genetic isolation. These locations had the poorest connectivity of tree cover with other sample locations. The cluster analyses revealed high membership values to a single cluster in each case (Q=0.88-0.97) while the pairwise *F*_ST_ values with other locations ranged from 0.112 to 0.227 in Mackay and from 0.043 to 0.180 in Brisbane. The period of isolation among these locations is of the order of 30-50 years (i.e. 10-20 generations). Genetic differentiation was apparent over a distance of 3 km in Brisbane and 8 km in Mackay, but was independent of geographic distance. The absence of isolation-by-distance in both landscapes is consistent with previous studies that have reported a breakdown of isolation-by-distance in fragmented landscapes [56,57]. Genetic drift within small, isolated populations may be so strong that allele frequencies drift independent of geographic distance. The Minnippi sites and Belmont Hills-Gateway-Mt Petrie sites clustered separately and pairwise *F*_ST_ values were >0.1. We found no evidence of contemporary gene flow between these clusters despite large sample sizes (n=147 and 63, respectively). For Padaminka in Mackay, we detected a high level of contemporary immigration. This may represent migration from an unsampled location or even the possibility of unrecorded releases from rehabilitation. However, this has not been sufficient to obscure the very strong signal of genetic isolation for this location. Among bobcats (*Lynx rufus*) and coyotes (*Canis latrans*) in southern California, frequent dispersal events were detected across highways but this did not prevent genetic differentiation [20]. Dispersal will only lead to gene flow if the disperser breeds with residents in its new location. 

 The second key feature was that locations with high levels of intervening tree cover clustered together. In Mackay, Weston and The Leap (3 km apart) clustered together (group 3; Q=0.60-0.78) and showed low genetic divergence (*F*_ST_<0.02). In Brisbane, the 3-km separation of Belmont Hills and Mt Petrie-Gateway has been exacerbated by the Gateway Freeway for 25 years. However, these sites clustered together (group 4; Q=0.68-0.84) and showed low divergence (*F*_ST_<0.05). Isolation has apparently not occurred because the intervening area has stepping stones of tree cover and road-side tree height along a road cutting would allow a glide crossing of the 40-m road gap. Likewise Karawatha and Kuraby, with a 20-m road gap between them for an equivalent period showed little differentiation (cluster group 3; Q=0.76-0.82; *F*_ST_<0.01). In the case of the Minnippi sites (cluster group 2; Q=0.92-0.97), where tree cover connection has been maintained, we detected recent migration from Minnippi Parklands to Minnippi East 800 m away. Contemporary migration up to 7 km has been detected in another species of gliding mammal (the greater glider, *Petauroides volans*) where tree cover was provided by exotic pine plantations [58].

The increasing genetic structure as habitat connectivity is broken down is consistent with studies on other taxa [8,21,59–62]. Ecological studies show a dramatic reduction in squirrel glider movements as canopy gaps increase beyond 50 m [27,63,64], so it is expected that larger disjunctions in habitat will greatly reduce dispersal. The spatial scale at which squirrel glider genetic structure became evident with the loss of intervening tree cover was 3-10 km. Evidence of ongoing genetic differentiation is also consistent with this spatial scale. In Brisbane, Belmont-Gateway-Mt Petrie showed stronger affinity with cluster 4 (Q=0.68-0.84) than with cluster 3 (Q=0.01-0.06) to which Kuraby-Karawatha, 10 km away, were aligned (Q=0.76-0.82). We detected no contemporary migration between these clusters (n=63 and 40, respectively). A substantial loss of tree cover between the Belmont-Gateway-Mt Petrie and the Kuraby-Karawatha cluster groups occurred 20 years before our sampling with the construction of the South East Freeway (M1 Motorway) and the Gateway Freeway that borders all of these remnants. This observation of increasing genetic structure also lends support to the hypothesis that the temporal scale for more complete differentiation may be approximately 30 years (i.e. 10 generations). Our results provide a snapshot of ongoing population genetic changes which are likely to become more extreme over time. 

### Genetic diversity

A consequence of genetic isolation is an increase in inbreeding and genetic drift [8,21,65–67]. Such effects will be exacerbated where population size is small and likely for any remnant <250 ha in area. Across our two study landscapes the locations most genetically differentiated (Padaminka, both Minnippi sites and Bracken Ridge) had the lowest measures of genetic diversity and highest levels of relatedness. Demographic studies confirm that these locations have small populations (Padaminka: <50 individuals [27],; Minnippi Parklands: 50-100 individuals [35],; Bracken Ridge: 50-70 individuals, D. Sharpe unpubl. data]. We also detected a higher than expected level of relatedness among samples from Slade Point and Belmont Hills which showed less genetic isolation. Belmont Hills is known to be characterised by a small population (<50 individuals; Goldingay unpublished data). We hypothesise that an increase in relatedness may precede other signatures of genetic isolation. Inbreeding can occur relatively quickly once populations become isolated [8,68]. 

It is unknown whether the loss of variation we detected at neutral genetic markers reflects a loss of adaptive variation, but there is empirical and theoretical support for this [69–73]. Irrespective of consequences of loss of evolutionary potential, loss of genetic diversity is predicted to have adverse consequences over time due to its impact on reproductive fitness and survival [22,68,73,74]. Contemporary migration among squirrel glider populations examined in our study appeared insufficient to prevent ongoing genetic drift and increasing levels of inbreeding. Thus, there is likely to be an increased risk of extinction associated with loss of genetic diversity at the Slade Pt, Padaminka, Minnippi Parklands, Minnippi East and Bracken Ridge locations in particular.

### Conservation implications

This study has particular implications for the conservation of vertebrates in urbanising landscapes where landscape change is most rapid. Our findings are consistent with another recent study [8] where population genetic changes induced by habitat loss and fragmentation occurred over small spatial (3-10 km) and temporal scales (20-30 years). Theory predicts that these changes are likely to be precursors to population collapse. Localised extinctions have been observed or inferred in studies of other taxa where habitat fragmentation has isolated small populations [4,5,7,66]. Thus, key management decisions for the conservation of biodiversity need to be made sooner rather than later because it will be immensely more expensive to repopulate landscapes once local extinction has occurred.

In the case of the squirrel glider, sensitivity to loss of habitat connectivity was previously assumed and led to some trials of engineered solutions such as tall wooden poles [75,76] and canopy rope-bridges [77–79] to restore habitat connectivity. Evidence is accumulating that these engineered solutions to loss of tree cover are successful for this species. Installation of glide poles at Padaminka in Mackay enabled squirrel gliders to cross between small habitat fragments separated by 75 m [27] whilst glide poles on a land-bridge have enabled squirrel gliders to cross a 60-m gap between our Karawatha and Kuraby sites in Brisbane [80]. Therefore, the tools to restore habitat connectivity for the squirrel glider and prevent local extinction are available. Genetic studies can be used to determine the success or otherwise of such interventions and their further development [81]. 

How common among taxa is fine-scale genetic malfunction likely to be? Evidence is accumulating that such effects will be expected for species of limited dispersal ability (particularly where constrained by habitat) and habitat specialists where the matrix is highly developed and contains pronounced barriers to movements such as wide freeways [e.g. 8, 14, 59, 61, 66, 67, 82, 83]. Studies on additional species are required to test these generalities. Further confirmation would allow land managers to target those species with life history attributes that predispose them to fine-scale effects of urbanisation. Species identified as vulnerable could then be used to guide efforts to restore and maintain landscape-level habitat connectivity for broader components of biodiversity.
